# Beyond Mesothelioma: From Diagnostic Biomarker to Therapeutic Opportunity—The Emerging Role of MTAP in Cancer

**DOI:** 10.3390/ijms27219653

**Published:** 2026-10-28

**Authors:** Anna Diluvio, Stefano Lucà, Lucia Iavarone, Valeria Masola, Alfonso Fiorelli, Floriana Morgillo, Giuseppe D’Abbronzo, Renato Franco

**Affiliations:** 1 Pathology Unit, Department of Mental Health and Physic and Preventive Medicine University of Campania “L. Vanvitelli”, 80138 Naples, Italylucia.iavarone@unicampania.it (L.I.); dabbronzogiuseppe@gmail.com (G.D.); 2 Thoracic Surgery Unit, Department of Cardio-Thoracic and Respiratory Sciences, Università degli Studi Della Campania “Luigi Vanvitelli”, 80138 Naples, Italy; 3 Medical Oncology, Department of Precision Medicine, University of Campania “L. Vanvitelli”, 80138 Naples, Italy

**Keywords:** MTAP, *CDKN2A*, mesothelioma, melanoma, predictive markers

## Abstract

Methylthioadenosine phosphorylase (MTAP) loss is an emerging biomarker in several human malignancies, mainly due to its frequent co-deletion with *CDKN2A* on chromosome 9p21. Beyond its diagnostic value, MTAP deficiency has important biological consequences, including metabolic reprogramming, immune microenvironment modulation, and increased dependency on PRMT5-related pathways. This review summarizes the current evidence on MTAP alterations across different tumor types. The role of MTAP immunohistochemistry as a practical surrogate for *CDKN2A* homozygous deletion is discussed, together with its advantages and interpretative limitations. In addition, the potential predictive relevance of MTAP loss for immunotherapy, antifolate-based treatments, and novel PRMT5-targeted strategies is explored. Overall, MTAP assessment represents a promising tool in modern diagnostic pathology and precision oncology, bridging morphology, molecular alterations, and therapeutic decision-making.

## 1. Introduction

The gene encoding the metabolic enzyme methylthioadenosine phosphorylase (*MTAP*) is located within the 9p21 chromosomal region, where it is closely linked to the tumor suppressor gene cyclin-dependent kinase inhibitor 2A (*CDKN2A*) and a cluster of interferon genes [1]. Because of this genomic proximity, homozygous loss of MTAP frequently co-occurs with deletions of *CDKN2A* and is observed in roughly 10% of human cancers overall [2]. The prevalence of *MTAP* deletion is particularly high in selected malignancies, including glioblastoma (~45%), mesothelioma (~36%), urothelial carcinoma (~26%), malignant peripheral nerve sheath tumors (~25%), pancreatic ductal adenocarcinoma (PDAC) (~22%), and non-small cell lung cancer (~15%) [2,3]. Loss of *MTAP* function—through gene deletion and/or absence of protein expression—has wide-ranging biological consequences, affecting metabolic homeostasis, epigenetic regulation, immune-related signaling pathways, and mechanisms governing cell motility and invasiveness [4,5,6,7,8,9,10]. Accumulating evidence supports a tumor-suppressive role for *MTAP*, with its loss contributing to cancer initiation and progression, aggressive tumor behavior, and unfavorable clinical outcomes. Mechanistically, MTAP deficiency has been linked to the dysregulation of key oncogenic drivers, such as the upregulation of the stemness-associated marker PROM1/CD133 and the kinase RIOK1, which foster tumor cell proliferation in glioblastoma and pancreatic cancer, respectively [4,5,6,7,8,9,10,11,12,13,14].

Despite these advances, the full spectrum of biological and clinical consequences associated with *MTAP* loss across different tumor types remains incompletely characterized and continues to be actively investigated. At present, *MTAP* status may be regarded as a potentially informative diagnostic adjunct within selected, complex pathological diagnostic algorithms.

Previous reviews have mainly addressed *MTAP* loss in thoracic tumors [15], across solid and hematological malignancies from an epidemiological perspective [16], or in terms of metabolic vulnerabilities and targeted therapeutic strategies [17]. The primary aim of the present review is to critically analyze the major issues surrounding the immunohistochemical assessment of MTAP—whose scoring criteria for positivity and loss currently remain largely operator- and center-dependent—and to discuss the strategies proposed to achieve an objective and reproducible evaluation. Furthermore, this review evaluates the extent to which MTAP expression can serve as a surrogate marker for *CDKN2A* deletion, offering significant cost savings. Finally, we explore the emerging predictive and therapeutic implications of MTAP loss, with a particular focus on poor-prognosis malignancies.

This review focuses on mesothelioma, melanoma, gastrointestinal cancers, central nervous system (CNS) tumors, and lung cancer, while also providing an overview of other malignancies such as neuroendocrine tumors and lymphomas. These tumor types were selected to offer a comprehensive scope, prioritizing those entities with the most promising research findings. The manuscript is organized as follows: Section 2 addresses mesothelioma, Section 3 covers melanoma, Section 4 discusses digestive tract tumors, Section 5 focuses on CNS tumors, Section 6 summarizes other tumor types, and Section 7 explores the predictive and therapeutic implications of MTAP loss.

## 2. Mesothelioma

Mesothelioma is a rare malignancy originating from the serosal membranes, with the pleura being the predominant site of manifestation [18]. The histopathological diagnosis of mesothelioma is highly complex, and, to date, the gold standard for diagnosing malignancy in a mesothelial proliferation is invasion into adjacent tissues and/or nodular tumor-forming growth pattern [19,20]. Unfortunately, these diagnostic features are not reliably captured in large biopsy specimens and are even less likely to be identified in small core biopsy samples and/or in cytological samples [20]. However, histomorphology alone could be insufficient for definitive diagnosis of mesothelioma [21,22,23,24].

Although neither BRCA1-associated protein 1 (BAP1) nor *CDKN2A* alterations confer absolute specificity for the diagnosis of malignant mesothelioma, ancillary studies can substantially aid the differential diagnostic workup [18,19]. Immunohistochemical (IHC) loss of BAP1 is highly specific for differentiating malignant mesothelioma from benign mesothelial proliferations [24,25,26,27,28,29,30,31,32,33,34]. BAP1 IHC loss is observed in only 55% of pleural mesotheliomas overall [34].

The *CDKN2A* homozygous deletion is diagnostic of malignancy in a mesothelial proliferation. The *CDKN2A* locus on chromosome 9p21 encodes p16 protein, but the immunohistochemical loss of p16, or related proteins p14 and p15, is not specific for *CDKN2A* homozygous deletion [31,35]. The deletion of this gene is evaluated by fluorescence in situ hybridization (FISH). Thus, *CDKN2A* FISH can be performed in challenging mesothelial lesions with retained BAP1, although *CDKN2A* deletion/alteration is reportedly detected by FISH in only 52–68% of mesotheliomas [24,27,35,36,37,38,39].

The longer turnaround times, higher costs, and limited availability of FISH compared with immunohistochemistry have driven interest in a reliable immunohistochemical surrogate for *CDKN2A* FISH [19]. Both genes, *CDKN2A* and *MTAP*, have been reported to be co-deleted in multiple malignancies as with malignant mesothelioma [16,19,35,36,40,41].

Early FISH-based studies demonstrated that up to 90% of pleural and peritoneal mesotheliomas harboring homozygous *CDKN2A* deletions also show concomitant loss of MTAP [35,36,42].

Krasinskas et al. sought to determine the prevalence of homozygous deletions of *CDKN2A* and/or *MTAP* in a series of peritoneal mesothelioma cases. In addition, they investigated the correlation between *CDKN2A* deletions identified by FISH and the loss of protein expression detected by immunohistochemistry. The results showed homozygous deletion of *MTAP* in nine cases, all of which also harbored *CDKN2A* deletions. Therefore, the correlation between *MTAP* and *CDKN2A* deletions was 100% [35].

As early as 2003, Illei et al. investigated *CDKN2A* deletion and *MTAP* co-deletion in pleural mesotheliomas using the FISH technique. Their analyses demonstrated that 74% of the examined cases harbored a homozygous deletion of *CDKN2A*, with *MTAP* being co-deleted in 91% of these. Moreover, no case showed *MTAP* deletion in the absence of concurrent *CDKN2A* deletion. The authors therefore concluded that homozygous deletion of *CDKN2A* occurs in most pleural mesotheliomas, with *MTAP* co-deleted in most of these cases [36].

A methodological caveat should be considered when interpreting these FISH-based data. The *CDKN2A*-specific FISH kits used in clinical practice have a probe set covering a genomic region of approximately 50 kb that extends from the *MTAP* gene to the *CDKN2A* gene. Notably, the investigations cited above predominantly rely on the Vysis LSI *CDKN2A*/CEP 9 probe (Abbott Molecular) [19,21,27,31,41,43,44,45,46,47,48]—or its commercial equivalent, the ZytoVision SPEC *CDKN2A* kit [49]—which currently stands as the most widely established standard dual-color commercial kit for fluorescence in situ hybridization (FISH) analyses across both histological and cytological specimens. This long probe (L-probe, ~222 kb) provides a sufficiently intense and stable fluorescent signal for clear microscopic evaluation; however, due to its size, it may fail to detect focal, small-scale homozygous deletions or specific breakpoints within the 9p21 region, for which a shorter probe (S-probe, ~57 kb) would be more suitable.

More recently, several studies have employed a monoclonal anti-MTAP primary antibody for the immunohistochemical detection of this enzyme. The results have been highly encouraging, as MTAP immunohistochemistry demonstrated acceptable sensitivity and specificity for identifying *CDKN2A* deletion and, consequently, for diagnosing mesothelioma [27,31,50].

Chapel et al. investigated MTAP immunohistochemistry (IHC) as a surrogate marker for *CDKN2A* deletion, which is traditionally evaluated by FISH, in a cohort of over one hundred pleural mesothelioma cases encompassing both malignant and benign lesions. The authors demonstrated that the sensitivity and specificity of MTAP IHC for identifying homozygous *CDKN2A* deletion, as well as interobserver reproducibility in MTAP scoring, were comparable across different specimen types, including cell blocks, biopsies, and surgical resections [19].

The results were highly encouraging: MTAP IHC proved to be a reliable surrogate marker for *CDKN2A* FISH, showing excellent interobserver concordance and interlaboratory reproducibility. Specifically, MTAP loss exhibited a sensitivity of approximately 80% and a specificity close to 100% for the detection of homozygous *CDKN2A* deletion. Interestingly, a rare case was reported with MTAP loss on IHC but a normal *CDKN2A* copy number by FISH. The underlying cause remains unclear, although it has been hypothesized to reflect either epigenetic silencing of the *MTAP* gene or a selective deletion of the *MTAP* locus without concurrent loss of *CDKN2A* [19,51].

Importantly, benign mesothelial proliferations with MTAP loss have not yet been documented in studies employing monoclonal anti-MTAP antibodies. Despite this unresolved aspect (i.e., the rare cases showing MTAP loss by IHC but a normal *CDKN2A* copy number by FISH), the authors conclude that MTAP IHC represents a robust and accurate diagnostic marker and advocate for its integration into the routine diagnostic work-up of malignant mesothelioma [19].

Previously, Churg et al. evaluated the effectiveness of combining MTAP and BAP1 immunohistochemistry (IHC) for distinguishing both epithelioid and sarcomatoid malignant pleural mesothelioma (MPM) from reactive mesothelial proliferations (RMPs), with additional analysis of their diagnostic performance in cell block samples. The results showed that MTAP IHC alone demonstrated high sensitivity for sarcomatoid MPM (80%), although this was slightly lower than that achieved with 9p21 FISH (93%). In cell block specimens, the combined use of BAP1 and MTAP IHC achieved a sensitivity of 89% in differentiating epithelioid MPM from reactive mesothelial cells, outperforming each test used individually, while the combination of BAP1 IHC and 9p21 FISH yielded the highest sensitivity (91.3%).

In conclusion, the combined application of MTAP and BAP1 IHC proved useful for the detection of malignant pleural mesothelioma, providing higher sensitivity compared with either marker alone or 9p21 FISH alone. Moreover, MTAP IHC alone showed good diagnostic performance for sarcomatoid MPM. These findings suggest that the combined assessment of MTAP and BAP1 by IHC may represent a valuable approach for differentiating malignant mesothelioma from reactive mesothelial proliferations, although the combination of BAP1 IHC and 9p21 FISH remains, at present, the most accurate ancillary diagnostic method [50].

According to Terra S et al., MTAP is frequently co-deleted in tumors that harbor homozygous deletions of the *CDKN2A* (p16) gene. In the context of malignant mesothelioma, loss of MTAP detected by immunohistochemistry is used as a surrogate marker for homozygous *CDKN2A* loss. In their study, the authors observed that although MTAP loss by immunohistochemistry is highly specific for *CDKN2A* loss in malignant mesothelioma, it is not perfectly sensitive [20].

In another study investigating the combination of immunohistochemistry (IHC) for MTAP and BAP1 in distinguishing malignant pleural mesothelioma (MPM) from reactive mesothelial hyperplasia (RMH), Hida T. et al. reported a sensitivity of 76.5% for the differential diagnosis between the two conditions. Notably, it was found that MTAP immunohistochemical analysis may be particularly useful in cases where BAP1 expression is retained. Therefore, the combined assessment of BAP1 and MTAP by IHC represents a valid, accessible, and cost-effective diagnostic approach for identifying mesothelioma, especially in laboratories where FISH analysis is not available. Furthermore, as also reported in other studies, no cases of reactive mesothelial proliferation showed loss or reduction in MTAP expression, thereby confirming a specificity of 100% for this marker [31].

Regarding pleural fluid cytology samples, Kinoshita Y et al. demonstrated that MTAP IHC can be a viable alternate assay in cell blocks for 9p21 FISH. The loss of MTAP IHC had a specificity of 100% for predicting the homozygous deletion of 9p21 detected by FISH, but approximately 32.1% of 9p21 homozygous deletion-positive cases detected by FISH demonstrated no loss of MTAP expression by IHC. The reason for this is that there are cases of mesothelioma in which *CDKN2A* is deleted, but the *MTAP* gene is not co-deleted [46]. Additionally, it has been documented that MTAP deletion alone is not observed in malignant mesothelioma, but it is possible the isolated homozygous deletion of *CDKN2A* [35,36]. Therefore, the authors of this study recommended to perform 9p21 FISH in cases in which MTAP loss is not detected by IHC, but the patient’s clinical presentation suggests the presence of clinical mesothelioma [21].

A major challenge in employing MTAP immunohistochemistry as a surrogate for FISH analysis of *CDKN2A* deletion lies in the lack of universally accepted criteria for defining signal positivity or negativity. Several factors contribute to this difficulty, including antibody variability arising from the use of different clones across laboratories, which are not always directly comparable. Additionally, tumor heterogeneity poses a significant obstacle, as certain neoplasms may exhibit partial or focal expression, complicating the establishment of a reliable cut-off. Finally, there is a lack of consensus on interpretative criteria, as it remains unclear whether a diffuse loss of tumor expression is sufficient or if a minimal residual fraction of positivity should be considered acceptable [17,43,52].

Although no gold standard has been established, most studies on MTAP report that tumor cell staining is regarded as positive, whereas the absence of staining in tumor cells, provided that an internal positive control is present, is interpreted as negative.

For example, in their study on MTAP and p16 immunohistochemical deficiency as surrogate markers of *CDKN2A/B* homozygous deletion in gliomas, Maragkou T. et al. considered MTAP, p16, and alpha-thalassemia/intellectual disability X-linked (ATRX) expression to be lost when tumor cells showed a total lack of staining, provided that appropriate internal positive controls were present. Based on these criteria, MTAP immunostaining was found to be homogeneous in tumor cells across all cases, with no evidence of partial staining. Only a very small number of cases exhibited slightly weak staining, yet cytoplasmic reactivity remained unequivocal. Interestingly, this minor variation appears to correspond to the *CDKN2A/B* hemizygous deletion, as confirmed by copy number variation (CNV) analysis [49].

Nuclear MTAP staining has been reported in a subset of cases but is inconsistent; the absence of nuclear MTAP alongside preserved cytoplasmic staining is considered indicative of MTAP retention [19,27,31].

In the study by Chapel et al., any case classified as “MTAP retained” by at least one scorer was subsequently reviewed by two pathologists (DBC, JS) [19], and the MTAP staining was designated as “faint” if there was consensus that the cytoplasmic staining in tumor cells was clearly weaker than that of the internal control.

These authors also emphasize that the interpretation of faint or negative MTAP staining in tumor cells relies on an unequivocal positive internal control, used to avoid overestimating *CDKN2A* deletion in cases where MTAP loss is artifactual due to pre-analytical issues, such as poor fixation, or failure of the immunohistochemical staining procedure. Clearly, the assessment of tumor staining relative to the internal control remains inherently subjective, and some cases are therefore unlikely to have a definitive interpretation. In such situations, consultation with additional pathologists and correlation with complementary studies—including *CDKN2A* FISH, if necessary—is recommended [19].

Terra S et al. in their study proposed evaluating the immunostaining pattern in a semiquantitative manner based on the proportion of tumor cells stained: negative if no appreciable staining was observed, focal if staining was present in less than 10% of tumor cells, patchy if 10–50% of tumor cells were positive, and diffuse if more than 50% of tumor cells were stained. However, at the conclusion of the interpretative analysis, all cases were ultimately classified either as negative or as showing staining in more than 50% of tumor cells. Therefore, in this context, the definitive criterion applied was that cytoplasmic expression in tumor cells is considered indicative of retained MTAP expression, whereas MTAP loss is defined as the absence of cytoplasmic staining in tumor cells despite a positive internal control [20] (Figure 1). Antibody clones, scoring criteria, internal controls and main pitfalls of MTAP IHC in the different tumor types are summarized in Table 1.

Since no gold standard is available to establish whether MTAP IHC can be considered a reliable surrogate predictor of *CDKN2A* deletion by FISH, orthogonal molecular assays may be helpful for validation. In this regard, Tian et al. reported a sensitive quantitative PCR assay for the detection of *CDKN2A* deletion for clinical practice, based on a 5.1-kb common deletion region within the *CDKN2A* gene identified across cancers [53]. Application studies on the prediction of prognosis in esophageal squamous cell dysplasia and gastric carcinoma metastasis have been promising [53]. Such approaches may help to overcome current limitations in the technologies available for the detection of *CDKN2A* deletion.

At present, the most advanced research efforts are focused on the characterization of *MTAP* deletion in mesotheliomas, predominantly those of pleural origin. However, it is important to note that this genomic alteration can be identified across a broad spectrum of neoplasms, which may therefore constitute a significant area of investigation both from a molecular diagnostic standpoint and for the development and optimization of targeted therapeutic strategies.

## 3. Melanoma

He LJ et al., in their study on *MTAP* deletion in nevi and melanomas, reported that homozygous deletion of *CDKN2A* is a frequent event in melanoma, whereas loss of p16 expression is not a reliable indicator of this alteration. Among the 45 nevi examined, p16 expression frequently exhibited a mosaic pattern, while *MTAP* expression was consistently preserved. In contrast, among the 70 melanomas analyzed, p16 loss was common, whereas *MTAP* loss was relatively rare [44].

Importantly, *MTAP* loss proved to be a highly specific marker of *CDKN2A* homozygous deletion, outperforming p16, which demonstrated numerous false positives. Moreover, *MTAP* expression was preserved in melanomas with single-copy *CDKN2A* deletion, unlike p16, further underscoring its superior diagnostic accuracy [44].

Behrmann et al. investigated the expression of the *MTAP* gene and its corresponding protein, considering the frequent co-deletion of *MTAP* with *CDKN2A* and its product p16. The authors analyzed nine human melanoma cell lines and primary human melanocytes and identified a marked down-regulation of MTAP mRNA expression in melanoma cell lines.

These findings were further validated by immunohistochemical analysis of 38 tissue specimens, including benign melanocytic nevi, primary melanomas, and melanoma metastases. Collectively, the data demonstrate a significant inverse correlation between MTAP protein expression and melanocytic tumor progression, characterized by a progressive reduction in MTAP immunoreactivity from benign melanocytic nevi to metastatic melanoma [51] (Figure 2).

Wild et al. analyzed MTAP expression in a large cohort of 315 benign and malignant cutaneous melanocytic tumors. Cytoplasmic MTAP expression was significantly reduced in primary malignant melanomas and melanoma metastases compared with benign nevi, while no significant difference was observed between primary melanomas and their matched metastatic lesions [54].

Within the spectrum of melanocytic lesions, a major diagnostic challenge is represented by MELTUMP (melanocytic tumors of uncertain malignant potential), which constitute a heterogeneous group of melanocytic proliferations lacking sufficient morphologic and biological criteria to allow a definitive classification as benign or malignant [55].

In this context, the mutational status of *CDKN2A* and MTAP has also been investigated. Loss of p16, encoded by the *CDKN2A* gene, represents a key event in melanoma pathogenesis [56]; however, p16 immunohistochemistry is characterized by limited specificity in borderline lesions due to frequently heterogeneous expression patterns. In contrast, MTAP immunohistochemistry shows very high specificity as a surrogate marker for *CDKN2A* homozygous deletion, albeit with lower sensitivity. Accordingly, complete and concordant loss of p16 and MTAP represents the most specific immunohistochemical surrogate for *CDKN2A*/*MTAP* homozygous deletion and strongly supports a diagnosis of melanoma or high-risk melanocytic neoplasm [44].

Conversely, partial or discordant staining patterns warrant further molecular investigations [55].

## 4. Digestive Tumors

The diagnosis of pancreatic lesions based on samples obtained via endoscopic ultrasound-guided fine-needle aspiration/biopsy (EUS-FNA/FNB) is often challenging due to the limited material available and its difficult morphological interpretation [57,58,59,60].

In this context, the use of immunohistochemical markers that reflect genetic alterations typical of PDAC becomes particularly relevant. Among these, the biallelic loss of *CDKN2A* is common; however, its assessment through p16 demonstrates limited sensitivity [14,19,27,45,46,61,62,63].

For this reason, the expression of MTAP has been investigated as a surrogate marker (Figure 3).

In a cohort of 136 pancreatic samples, complete loss of MTAP expression was observed in approximately 30% of PDAC cases and in none of the benign samples, indicating high specificity. Moreover, cases classified morphologically as “atypical” or “suspicious” showing MTAP loss were subsequently confirmed as PDAC, highlighting the value of this marker in the interpretation of ambiguous findings. In samples with available next-generation sequencing (NGS) data, MTAP loss consistently correlated with *CDKN2A* deletion.

Overall, MTAP immunohistochemistry emerges as a valuable complementary tool in small EUS-obtained samples, enhancing diagnostic confidence and improving the distinction between malignant and non-neoplastic lesions, particularly in cases with challenging morphological features [64].

Given that *CDKN2A* is inactivated in approximately 40% of pancreatic tumors through homozygous deletion, while in an additional 40% of cases the loss of one allele is associated with intragenic mutations and in about 15% of patients inactivation occurs via promoter hypermethylation [65,66], together with the well-documented co-deletion with MTAP, Lucà et al. evaluated MTAP expression status by immunohistochemistry (IHC) in the diagnostic setting of PDAC, analyzing its correlation with *CDKN2A* deletion assessed by FISH and NGS, with particular emphasis on small histological samples.

A total of 29 biopsy specimens were analyzed, including cases classified as malignant, suspicious, and benign. Among cases diagnosed as PDAC, loss of MTAP expression was observed in 25% of cases (6/24). Diagnostic performance analysis demonstrated a high positive predictive value (PPV) of 100%, contrasted by a very low negative predictive value (NPV) of 7%, with limited sensitivity (31.5%) and high specificity (100%) for the diagnosis of PDAC on EUS-FNAB specimens.

A non-negligible proportion of discordant cases between MTAP IHC and *CDKN2A* FISH was observed, whereas a stronger concordance emerged between NGS results and immunohistochemistry. Notably, NGS demonstrated a markedly higher sensitivity compared with FISH, likely attributable to the limited cellularity of the analyzed samples.

In conclusion, these data indicate that *MTAP* loss is a frequent event in PDAC and that homozygous deletion of *CDKN2A* can be reliably predicted by loss of MTAP immunohistochemical expression [47].

The impact of MTAP alterations in gastrointestinal (GI) cancers remains poorly characterized to date. A notable contribution comes from the study by Giancluca Mauri et al., which investigated the prevalence, clinicopathological features, and prognostic significance of MTAP loss in GI tumors.

In their analysis, gene loss emerged as the most frequent *MTAP* alteration (9.4%), occurring almost invariably in co-deletion with *CDKN2A/B* (97.7%). Of particular interest is the association with microsatellite stability, as MTAP loss was identified almost exclusively in microsatellite-stable (MSS) tumors, suggesting a potential biological relationship between these molecular events.

From a distributional standpoint, the highest prevalence of *MTAP* loss was observed in biliopancreatic and gastro-esophageal cancers, with rates of 20.5% and 12.7%, respectively, whereas the phenomenon appeared far less common in colorectal tumors [67].

## 5. Central Nervous System (CNS) Tumors

The role of MTAP immunohistochemistry has also been investigated in specific histotypes of brain tumors, as homozygous deletion of *CDKN2A* represents a key prognostic biomarker in isocitrate dehydrogenase (IDH)-mutant diffuse gliomas [68,69,70,71,72,73].

In this context, loss of MTAP staining has been proposed as a potential surrogate for *CDKN2A* deletion. In a study by Kaishi Satomi et al., the accuracy of MTAP immunoreactivity as a proxy for *CDKN2A* homozygous deletion in adult diffuse gliomas was evaluated [48].

MTAP immunohistochemistry (clone EPR6893) was assessed in 178 cases, including IDH-mutant astrocytomas, IDH-mutant oligodendrogliomas, and IDH-wildtype glioblastomas. Loss of MTAP expression demonstrated good predictive performance for *CDKN2A* homozygous deletion (HD) in IDH-mutant astrocytomas (sensitivity 88%; specificity 98%) and IDH-wildtype glioblastomas (sensitivity 89%; specificity 100%), whereas it proved less reliable in IDH-mutant oligodendrogliomas [48].

Overall, MTAP staining was easily interpretable in 61% of cases. These findings suggest that MTAP loss may serve as a useful surrogate for *CDKN2A* homozygous deletion in IDH-mutant astrocytomas and IDH-wildtype glioblastomas, while also providing significant prognostic value in IDH-mutant astrocytomas [48].

Maragkou et al. evaluated the use of MTAP and p16INK4a immunohistochemistry as surrogate markers for homozygous deletion of the *CDKN2A/B* locus across different glioma subtypes and grades. In a cohort of 100 diffuse and circumscribed gliomas, MTAP and p16 expression was correlated with *CDKN2A/B* status. Complete loss of MTAP expression demonstrated excellent diagnostic performance for detecting *CDKN2A/B* homozygous deletion, with a sensitivity of 100% and a specificity of 97%, whereas p16 showed lower diagnostic accuracy. In the few discordant cases between immunohistochemistry and copy number analysis, fluorescence in situ hybridization (FISH) confirmed the presence of homozygous deletion [49].

Homozygous deletion of the *CDKN2A/B* locus represents a major adverse prognostic factor in diffuse gliomas, irrespective of IDH mutation status. Furthermore, loss of MTAP expression was significantly associated with reduced overall survival in IDH-mutant astrocytomas and oligodendrogliomas, and to a lesser extent in IDH-wild-type gliomas. Overall, MTAP immunohistochemistry represents a reliable, rapid, and cost-effective tool for the indirect identification of *CDKN2A/B* homozygous deletion in gliomas, whereas the use of p16INK4a as a surrogate marker should be interpreted with caution [74,75].

**Table 1:** Summary of MTAP immunohistochemistry in the tumor types discussed.

Ref.	Study	Site/Tumor (Specimen)	Antibody Clone	Scoring Method	Internal Control	Reported Limitations
[19]	Chapel 2020	Pleura—mesothelioma (tissue, 5 centers)	2G4 and 42-T (different laboratory protocols)	Loss = absence of cytoplasmic staining; faint staining (weaker than the control) classified as loss	Lymphocytes, endothelial cells, fibroblasts	Interobserver disagreement was concentrated in faint cases; nevertheless, κ = 0.85 between observers and 0.77–0.89 between laboratories
[20]	Terra 2022	Pleura/lung—sarcomatoid mesothelioma vs. sarcomatoid carcinoma (tissue)	2G4 (Abnova)	Binary assessment of loss (yes/no) on whole sections	Tumor-associated inflammatory or mesenchymal cells	Loss was also frequent in sarcomatoid carcinoma (50%): MTAP is not helpful in this differential diagnosis
[21]	Kinoshita 2018	Pleura—effusion (cell block)	2G4 (Abnova), 1:100	Percentage of mesothelial cells with retained staining among ≥500 cells; 50% cutoff	Histiocytes, lymphocytes, fibroblasts, pneumocytes, endothelial cells	Distinguishing isolated mesothelioma cells from reactive mesothelial cells and histiocytes is difficult; dual EMA/MTAP staining is required
[27]	Berg 2018	Pleura—epithelioid mesothelioma vs. reactive mesothelium (TMA)	2G4 (Abnova), 1:100, pH 9	Loss of cytoplasmic staining; in positive cases, loss involved nearly 100% of cells	Inflammatory and stromal cells	TMA may underestimate heterogeneity; positivity criteria differed from those of Hida 2017
[31]	Hida 2017	Pleura—mesothelioma vs. reactive mesothelial hyperplasia (tissue)	2G4 (Abnova), 1:100, pH 9	Staining intensity ≥ internal control = retained; percentage cutoff derived from ROC curves	Inflammatory and stromal cells	Cohort-specific ROC cutoff, not directly transferable
[41]	Berg 2020	Pleura—effusion (cytology)	2G4 (Abnova), 1:100, pH 9	Complete loss of cytoplasmic staining; partial loss quantified as a percentage		Partial loss in 3 cytology specimens (40–80% positive cells) with 100% positivity in the corresponding histology: uncertain significance
[43]	Febres-Aldana 2024	Pleura—diffuse pleural mesothelioma (tissue, 56 cases)	EPR6893 (Abcam) 1:1000 vs. 1813 (Novus) 1:2500	Retained = cytoplasmic intensity ≥ control; equivocal = faint blush, heterogeneity, or inadequate control	Stromal and/or immune cells; mandatory	EPR6893 was equivocal in 34% (blush, weak internal control, heterogeneity); no equivocal cases with 1813. Reference to be verified
[45]	Chapel 2021	Pleura—mesothelioma (tissue)	2G4 (Abnova)	Loss of cytoplasmic staining; faint staining = markedly weaker than control	Not reported	Homozygous MTAP/*CDKN2A* co-deletion was present in only 13%; inconsistent correlation with heterozygous MTAP deletion
[46]	Hamasaki 2019	Pleura—effusion (cytology)	2G4 (Abcam)	Loss of cytoplasmic staining	Not reported	Complete methodological details unavailable
[64]	Yu 2024	Pancreas—PDAC (EUS-FNA cell block and core biopsy)	2G4 (Abcam)	Loss = absence of cytoplasmic and nuclear staining with positive non-neoplastic cells; equivocal category	Non-neoplastic cells	Limited sensitivity (~28%); equivocal cases; NGS available in only 13 cases
[47]	Lucà 2025	Pancreas—PDAC (EUS-FNAB)	2G4 (Abcam), Ventana BenchMark ULTRA	Yu criteria: complete cytoplasmic and nuclear loss; equivocal = weak staining in most cells (<20% weakly positive)	Non-neoplastic cells	Only 29/63 specimens (46%) adequate; 6/27 equivocal (22%); 40.7% MTAP–FISH discordance in paucicellular specimens
[67]	Mauri 2025	Gastrointestinal tract (NGS/TCGA; IHC in selected cases)	Rabbit polyclonal anti-MTAP, Proteintech (Tucson, AZ, USA)	Presence/absence of protein expression	Not reported	MTAP point mutations generally did not result in protein loss; only MTAP-CDKN2B truncation resulted in negative IHC
[49]	Maragkou 2023	Central nervous system—gliomas (tissue; ngTMA)	2G4, 1:200	Complete loss in tumor cells	Endothelial cells	Two MTAP-negative cases without homozygous deletion on CNV plots were subsequently confirmed by FISH, highlighting limitations of the molecular reference standard
[48]	Satomi 2021	Central nervous system—diffuse gliomas (178 specimens)	EPR6893 (Abcam) 1:1000; compared with 2G4 1:200	Loss of cytoplasmic staining; unevaluable if the internal control fails to stain	Endothelial cells, lymphocytes	Weak control required examination of more than one high-power field in 28% of cases; distinguishing tumor from reactive glia and interpreting perinuclear halos were difficult; 2G4 yielded stronger staining (66% of straightforward cases)
[44]	He 2025	Skin—nevi and melanomas (tissue)	2G4 (Abnova); p16 E6H4 (Ventana)	Binary: loss = complete absence of cytoplasmic staining in melanocytes; mosaic pattern recorded separately	Non-neoplastic cells (stromal, inflammatory)	Monoallelic *CDKN2A* deletions resulted in retained or mosaic MTAP staining; sensitivity for *CDKN2A* homozygous deletion was 41%
[54]	Wild 2006	Skin—nevi, melanomas, metastases (TMA)	Rabbit polyclonal anti-MTAP antibody	Semiquantitative cytoplasmic intensity score 0–2 (0 = negative, 1 = weak, 2 = strong); weak = positive	Not reported	TMA samples represented only a small area of each tumor; therefore, even weak staining was counted as expression
[12]	Su 2014	Lung—NSCLC (TMA, 99 cases)	Rabbit polyclonal anti-MTAP antibody	Low vs. high expression (dichotomized)	Not reported	Prognostic expression score rather than a loss-of-expression score; not comparable with diagnostic studies

## 6. Other Tumor Types

In their study, N. Gorbokon et al. emphasize that although neuroendocrine tumors (NETs) and hematological malignancies are among the neoplasms displaying the most frequent loss of MTAP expression on immunohistochemical examination—reaching up to 80% of cases in NETs of various sites, 50% in Hodgkin lymphoma, and up to 14% in non-Hodgkin lymphomas and involving a significant fraction of T-cell lymphomas—they almost completely lack chromosomal deletions at the 9p21 locus [15].

Because the healthy precursor cells from which these tumors originate (such as normal lymphocytes or pancreatic islet cells) clearly express the MTAP protein, the absence of the protein in neoplastic tissues is attributable neither to low baseline levels in their cells of origin nor to a physical loss of the gene [15]. Instead, as confirmed by parallel studies cited in the literature, the downregulation and subsequent loss of MTAP protein in lymphomas and neuroendocrine tumors appear to stem from an epigenetic mechanism—specifically, hypermethylation of the *MTAP* gene promoter—which silences its expression without altering DNA structure [76].

Over the last two decades, the diagnostic and prognostic utility of methylthioadenosine phosphorylase (MTAP) expression in non-small cell lung carcinoma (NSCLC) has been increasingly investigated. In a pioneering study, Sandhu et al. evaluated the feasibility of immunohistochemistry (IHC) as a reliable, reproducible, and practical diagnostic tool to detect MTAP protein loss across a cohort of 75 NSCLC specimens. Their findings established IHC as an accessible surrogate method for identifying MTAP deficiency in tumor tissues, laying the groundwork for its application as both a biomarker and a rationale for targeted metabolic therapies [77].

Subsequently, Su and colleagues expanded upon these observations by investigating a large NSCLC cohort using tissue microarrays (TMAs). Their work demonstrated that diminished or lost MTAP expression, assessed immunohistochemically using a polyclonal rabbit anti-MTAP antibody (Proteintech Group), serves as an independent negative prognostic marker associated with significantly worse patient outcomes. Together, these landmark studies provided early clinical evidence supporting the routine evaluation of MTAP status by IHC in NSCLC, highlighting its dual significance as a prognostic factor and a key feature for novel therapeutic stratification [12].

## 7. Predictive Role

The identification of MTAP deletion is of relevance beyond its diagnostic value alone. Currently, a growing body of research is investigating the role of MTAP not only as a predictive biomarker of response to selected oncological therapies, but also as a potential therapeutic target, exploiting the functional consequences associated with the loss of protein expression [78].

### 7.1. MTAP Loss as a Predictive Biomarker of Response to Immunotherapy

Emerging evidence suggests that *MTAP* deletion may be associated with reduced efficacy of immune checkpoint inhibitor (ICI) therapy, thus representing a potential negative predictive biomarker [2,79]. In support of this hypothesis, Han et al. demonstrated that homozygous deletion of the 9p21 chromosomal region correlates with unfavorable clinical outcomes in patients receiving anti-programmed death-ligand 1 (PD-L1) monotherapy, independently of PD-L1 expression levels and tumor mutational burden (TMB) [2]. In non-small cell lung cancer (NSCLC), this association appeared more pronounced in patients treated with ICI monotherapy compared with those receiving combined immunotherapy and chemotherapy [79].

The biological mechanisms underlying this negative predictive effect have not yet been fully elucidated; however, accumulating evidence indicates that loss of MTAP contributes to the development of an immunologically “cold” tumor microenvironment. This effect is thought to be mediated by the accumulation of 5′-methylthioadenosine (MTA) within the tumor microenvironment, which suppresses the function of CD8^+^ and CD4^+^ T lymphocytes through inhibition of protein arginine methyltransferase 5 (PRMT5) and activation of adenosine receptors [2,80,81]. An additional mechanism may involve the co-deletion of the type I interferon (IFN) gene cluster, which occurs in approximately half of all 9p21.3 deletions [2]. Given the essential role of type I interferons in inflammation and immune regulation, their loss may further promote immune evasion and resistance to ICI therapy [82,83,84].

### 7.2. MTAP Deficiency and Antifolate-Based Chemotherapy

With respect to the use of conventional chemotherapeutic agents, another therapeutic approach of particular interest in MTAP-deficient tumors involves the use of antifolate agents, including pemetrexed. Given the essential role of MTAP in the adenine salvage pathway [85,86], neoplasms harboring MTAP deletion exhibit increased susceptibility to synthetic lethality-based strategies targeting de novo purine biosynthesis, particularly adenine synthesis [85]. Indeed, a retrospective analysis demonstrated that patients with MTAP-deficient lung adenocarcinoma experienced significantly improved responses to combination therapy with pemetrexed and carboplatin compared with patients with MTAP-proficient tumors [85]. Furthermore, in a small phase II clinical trial (NCT02693717), patients with MTAP-deficient metastatic urothelial carcinoma showed a higher overall response rate to pemetrexed than those with MTAP-positive tumors [85]. Overall, the study included 21 patients with metastatic urothelial carcinoma. In the historical cohort (*n* = 14), all MTAP-deficient patients achieved an objective response rate (ORR) of 100% (4/4), compared to 10% (1/10) in MTAP-proficient patients, 80% of whom (8/10) exhibited tumor growth from baseline as best response. In the prospective cohort (NCT02693717, *n* = 7), which comprised exclusively MTAP-deficient patients, the ORR (primary endpoint) was 42% (3/7) [85].

### 7.3. Therapeutic Strategies Targeting MTAP-Related Vulnerabilities

Novel therapeutic approaches are emerging that specifically exploit the loss of MTAP and its impact on tumor cell metabolism. In particular, MTAP-deficient cancer cells are highly dependent on residual PRMT5 activity, and further accumulation of intracellular 5′-methylthioadenosine (MTA) selectively compromises cell viability in MTAP-deficient tumor lines [4]. This metabolic vulnerability makes MTAP-deficient tumors ideal candidates for synthetic lethality-based therapeutic strategies [5]. In this context, next-generation MTA-dependent PRMT5 cooperative inhibitors selectively target the enzyme under conditions of elevated MTA, thereby enhancing antitumor activity while sparing MTAP-proficient normal cells [4,87,88].

For instance, MRTX1719 has shown high selectivity for PRMT5 in murine models, human tumor cell lines, and first-in-human clinical studies [89,90]. Similarly, AMG 193, another MTA-dependent PRMT5 cooperative inhibitor, has been evaluated in patients with advanced solid tumors harboring MTAP deletions in a first-in-human clinical trial (NCT05094336) [91]. Preliminary data recently reported indicate a favorable safety profile for AMG 193 [91]. Currently, several clinical trials are ongoing to evaluate novel PRMT5 inhibitors across a wide spectrum of advanced solid malignancies [92].

Consequently, all these promising advances in MTAP-targeted therapies underscore an increasing need for diagnostic strategies to detect MTAP that are reliable, readily implementable, and cost-effective across a wide range of tumor types [78].

## Figures and Tables

**Figure 1:**
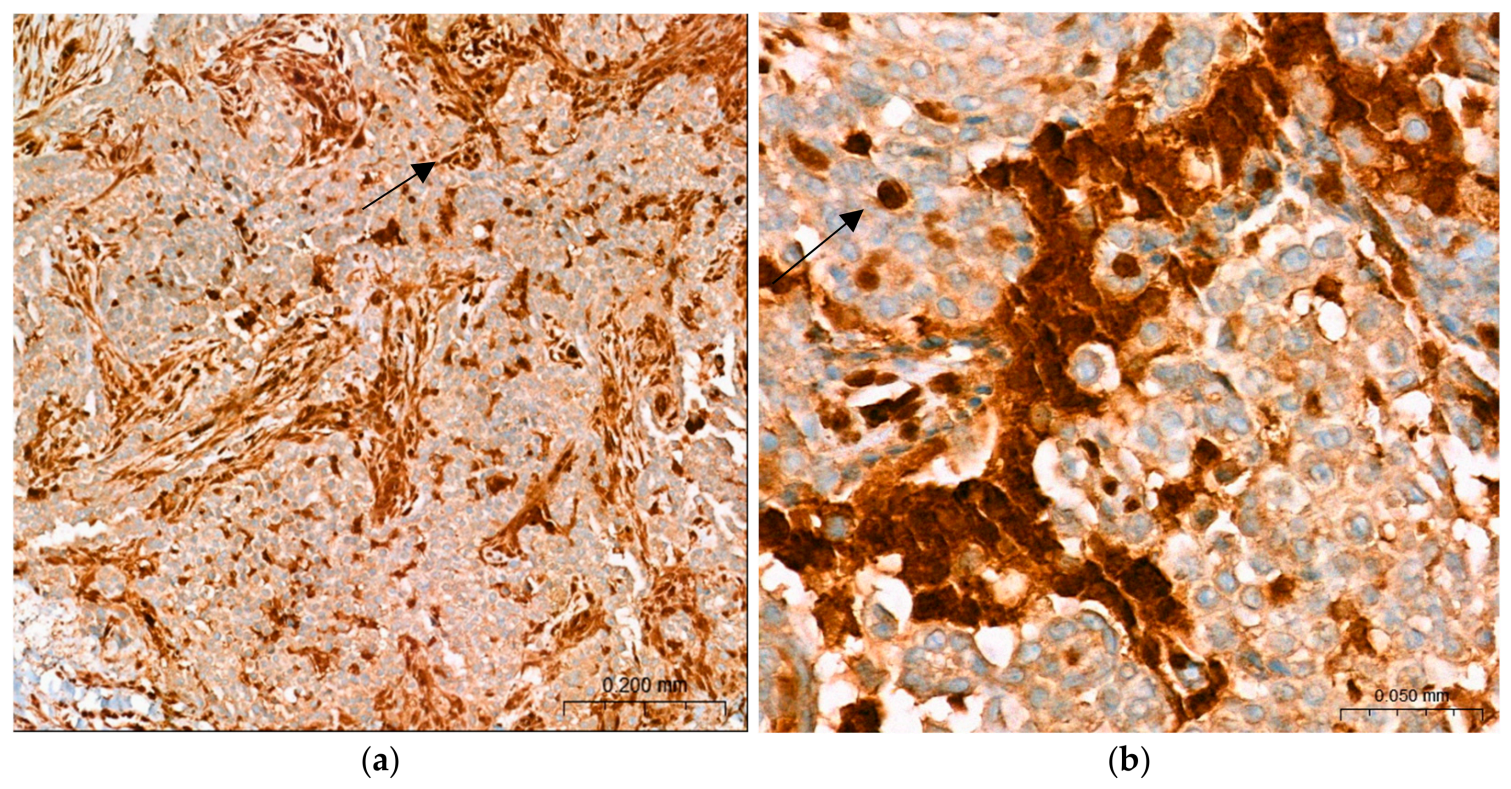
Mesothelioma: MTAP loss of expression: (**a**) 10× magnification; (**b**) 40× magnification. Arrows indicate the internal positive control. MTAP loss was defined as complete absence of cytoplasmic staining in tumor cells with preserved internal positive control 80% of tumor cells. Scale bars: (**a**) [200 µm]; (**b**) [60 µm].

**Figure 2:**
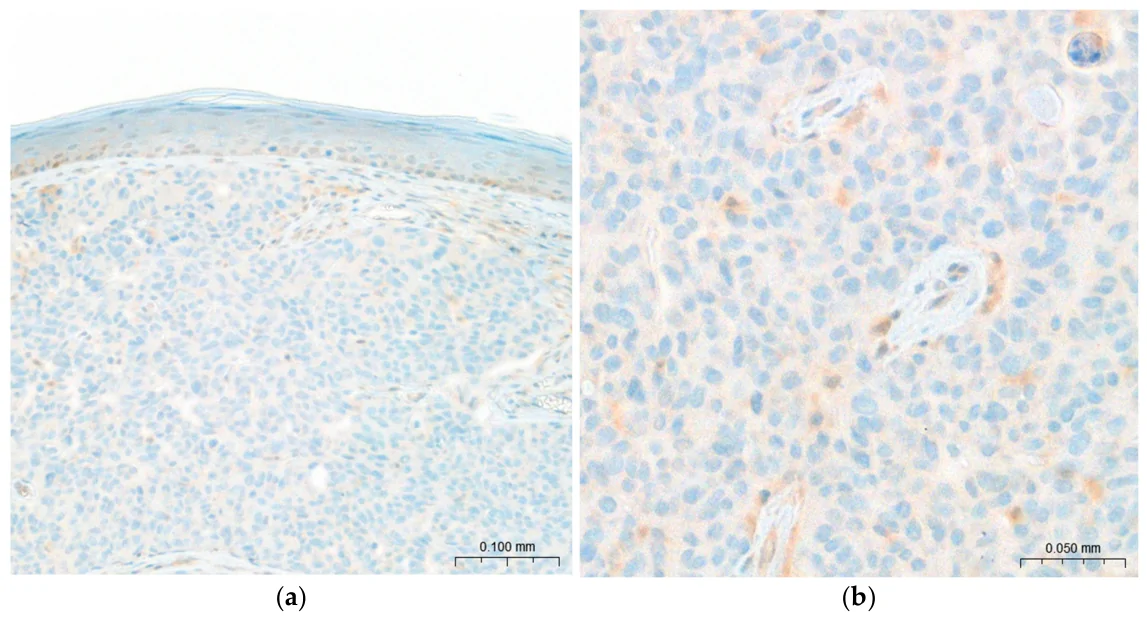
Melanoma: MTAP loss of expression: (**a**) 20× magnification; (**b**) 40× magnification. MTAP loss was defined as the complete absence of cytoplasmic staining in tumor cells with preserved internal positive control; 100% of tumor cells. Scale bars: (**a**) 100 µm; (**b**) 50 µm.

**Figure 3:**
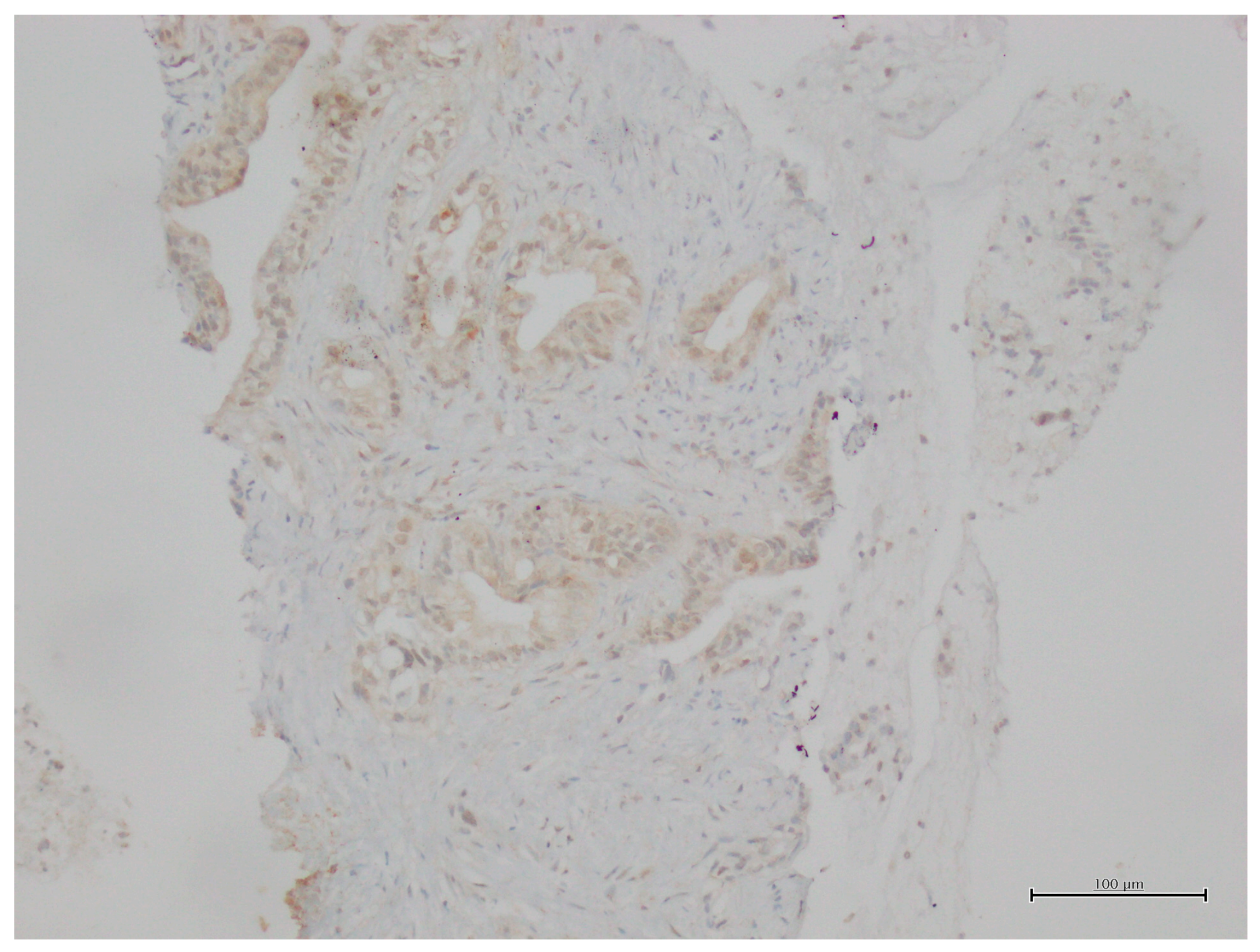
Pancreatic carcinoma: MTAP loss of expression MTAP loss was defined as a complete absence of cytoplasmic staining in tumor cells with preserved internal positive control; 100% of tumor cells. Scale bars: 100 µm.

## Data Availability

No new data were created or analyzed in this study. Data sharing is not applicable to this article.
